# Supplementary material for: Overexpression of SgDREB2C from Stylosanthes guianensis Leads to Increased Drought Tolerance in Transgenic Arabidopsis
Source: Int J Mol Sci. 2022 Mar 24;23(7):3520. doi: 10.3390/ijms23073520 (PMC8998575; doi:10.3390/ijms23073520)
Supplement: Supplementary file 1 [file ijms-23-03520-s001.zip › ijms-1617230-supplementary.pdf]

Table S1. Primer sequences for RT-qPCR.

Gene	Forwoard primer 5'-3'	Reverse primer 5'-3'
AtAPX1	ATCTCTTATGCGGACTTA	GCTACAACATCAACTCTC
AtAPX2	CAACTACCAACCGACAAG	GAAGAAGGCATCCTCATC
AttAPX	GCTACTAAATCTTCATCTTCAG	ATGCCAACCTAATCTAACC
AtCAT1	TTCCCTGACATGGTCCATGC	GCCTTCCATGTGCCTGTAGT
AtCAT2	GCCGATACTCAGAGACACCG	CTCAGCATGACGAACCTGGT
AtCAT3	ACATGGAGGGTTTCGGTGTC	TTAGTGGCGTGGCTGTGATT
AtSOD1	ACAGCAGTGAGGGTGTTACG	GGTGTCACCAAGAGCATGGA
AtCOR15A	ATGGCGATGTCTTTATCAGGAGC	TGACGGTACCAACACCACTCTG
AtCOR47	CAACAGCTCTTCTTCCTCTTCG	CCTTTCTTATCTTCCTCTCCTTCA
AtDREB2C	GAGGATTGTAGCGATGAA	CAGCCAAGTTATGTTCTTC
AtKIN1	TGTCAGAGACCAACAAGAATGCC	TGTCCAGCAGAACATTGCTCTTC
AtRD29A	GTTACTGATCCCACCAAAGAAGA	GGAGACTCATCAGTCACTTCCA
SgDREB2C	TTCAGACACAACTACTATATC	AAGCAACAGAGTCATAAG
AtACTIN	TGTGCCAATCTACGAGGGTTT	TTTCCCGCTCTGCTGTTGT
SgACTIN	GTTCTTCTCCAGCCATCT	CCTTGCTCATACGGTCAG
